# Physician Perspectives on Addressing Anti-Black Racism

**DOI:** 10.1001/jamanetworkopen.2023.52818

**Published:** 2024-01-24

**Authors:** Crystal E. Brown, Arisa R. Marshall, Kristine L. Cueva, Cyndy R. Snyder, Erin K. Kross, Bessie A. Young

**Affiliations:** 1 Cambia Palliative Care Center of Excellence at UW Medicine, Seattle, Washington; 2 Division of Pulmonary, Critical Care, and Sleep Medicine, Department of Medicine, University of Washington, Seattle; 3 Department of Bioethics and Humanities, School of Medicine, University of Washington, Seattle; 4 Department of Medicine, Center for Health Workforce Studies, School of Medicine, University of Washington, Seattle; 5 Department of Family Medicine, University of Washington, Seattle; 6 Division of Nephrology, Department of Medicine, University of Washington, Seattle; 7 Justice, Equity, Diversity, and Inclusion Center for Transformational Research, Office of Healthcare Equity, University of Washington, Seattle

## Abstract

**Importance:**

Uncertainty remains among clinicians regarding processes to address and resolve conflict around anti-Black racism.

**Objective:**

To elicit clinicians’ perceptions of their role in addressing concerns about anti-Black racism among Black patients with serious illness as well as their families.

**Design, Setting, and Participants:**

In this qualitative study, one-on-one semistructured interviews were conducted with 21 physicians at an academic county hospital between August 1 and October 31, 2022. Participants were provided clinical scenarios where anti-Black racism was a concern of a patient with serious illness. Participants were asked open-ended questions about initial impressions, prior similar experiences, potential strategies to address patients’ concerns, and additional resources to support these conversations. A framework based on restorative justice was used to guide qualitative analyses.

**Main Outcomes and Measures:**

Perspectives on addressing anti-Black racism as described by physicians.

**Results:**

A total of 21 medical subspecialists (mean [SD] age, 44.2 [7.8] years) participated in the study. Most physicians were women (14 [66.7%]), 4 were Asian (19.0%), 3 were Black (14.3%), and 14 were White (66.7%). Participants identified practices that are normalized in clinical settings that may perpetuate and exacerbate perceptions of anti-Black racism. Using provided scenarios and personal experiences, participants were able to describe how Black patients are harmed as a result of these practices. Last, participants identified strategies and resources for addressing Black patients’ concerns and facilitating conflict resolution, but they stopped short of promoting personal or team accountability for anti-Black racism.

**Conclusions and Relevance:**

In this qualitative study, physicians identified resources, skills, and processes that partially aligned with a restorative justice framework to address anti-Black racism and facilitate conflict resolution, but did not provide steps for actualizing accountability. Restorative justice and similar processes may provide space within a mediated setting for clinicians to repair harm, provide accountability, and facilitate racial healing.

## Key Points

**Question:**

What are physicians’ perceptions of their role in perpetuating, addressing, and providing accountability for anti-Black racism when treating Black patients with serious illness?

**Findings:**

In this qualitative study of 21 physicians, participants identified practices that may perpetuate and exacerbate perceptions of anti-Black racism. Participants identified strategies and resources for addressing patient concerns and facilitating conflict resolution, but they stopped short of promoting personal or team accountability for anti-Black racism.

**Meaning:**

This study suggests that processes that promote accountability, such as restorative justice, may provide space within a mediated setting for clinicians to repair harm, provide accountability, and facilitate racial healing.

## Teaser

This qualitative study examines clinicians’ perceptions of their role in addressing concerns about anti-Black racism among Black patients with serious illness as well as their families.

## Introduction

Black patients with serious illness are more likely to experience higher-intensity care, high symptom burden, and poor quality of life as they near the end of life.^1,2^ Conventional research approaches to these inequities often point toward personal attributes of Black patients and families without interrogating the structures or practices that perpetuate them.^3^ High-quality communication about serious illness focuses on eliciting patients’ values to promote goal-concordant care during the shared decision-making process.^4,5,6,7^ However, Black patients experience structural barriers and racial traumas that negatively affect their emotional well-being, perpetuate poor perceptions about the medical system, and prevent them from accessing fair and equitable care over the course of their lives.^8,9,10,11,12,13^ For example, Black patients experience unconscious bias, lack of physician diversity, and poor-quality communication.^14,15,16^ Even during serious illness and as they approach the end of life, Black patients, especially those with multiple marginalized identities (eg, experiencing addiction or being unhoused), continue to experience discrimination and microaggressions from health care workers (HCWs), negatively affecting perceptions of their communication with clinicians and the shared decision-making process.^17^ Such studies suggest that racist experiences and inequitable communication during serious illness adds to the accumulation of racism experiences that patients of racial and ethnic minority populations experience over the course of their lives.^13,18^

A qualitative study of medical subspecialists was recently conducted to inform the development of a clinician-facing component of a dual-facing program to address the distress of racism experienced by Black patients with serious illness.^19^ The purpose of that study was to elicit physician-identified resources to broadly improve conversations about anti-Black racism. Participants identified additional resources needed by both patients and clinicians to better facilitate discussions around anti-Black racism and other equity-based concerns. Participants also described elements of communication that align with restorative justice processes (RJPs), especially when participants described resolving conflicts where bias, discrimination, and racism were important concerns of patients and families. Restorative justice processes are structured processes based in Indigenous practices that allow community members to come together to address harmful behavior and create a path toward accountability and repair.^20^ Restorative justice processes have been used in multiple settings including schools, criminal justice, correctional and mental health facilities, and foster homes.^21,22,23^ Rather than formal investigatory processes that focus on whether a rule or policy was violated, RJPs respond to reports of harm by allowing for a safe, facilitated space for affected persons to tell offenders how they were affected and allows for support persons to provide additional, holistic descriptions of the effect of the harm.^24^ Moreover, affected persons play an active part in deciding how harm can be repaired, giving the opportunity for offenders to repair relationships and restore their standing in their community. These processes allow for the manifestation of the 5 guiding principles of restorative justice: relationships to be mended, fostering of mutual respect, encouragement of alleged offenders to assume responsibility for the harm they have caused, repair of harm to the fullest extent possible, and reintegration of offenders back into the community (ie, 5Rs framework). We hypothesized that RJPs could be used in the health care setting to resolve conflicts in which racism and other equity-based concerns were the center of patient-clinician conflict (Figure).^20,25^

**Figure.**
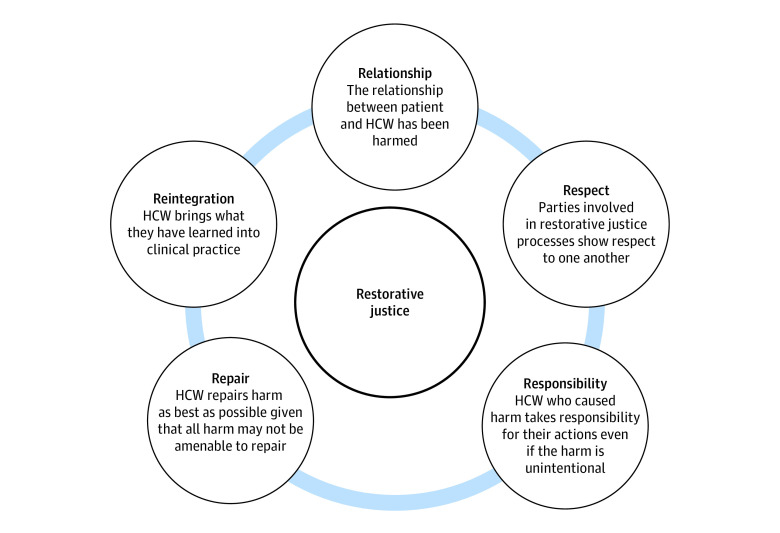
The 5Rs of Restorative Justice Used in the Health Care Setting to Promote Accountability and Repair the Harms of Anti-Black Racism HCW indicates health care worker.

In this study, we report the results of a secondary qualitative analysis of interviews with medical subspecialists experienced with caring for patients with serious illness to explore perspectives around conflict resolution. Secondary qualitative analysis allows us to investigate a research question that differs from the original research question by using existing data that address sensitive topics around racism, communication, and accountability.^26,27^ Using a coding framework based on RJPs, our goals were to further understand physician-identified needs and resources to resolve conflict, address anti-Black racism and other inequities, improve accountability, and promote racial healing. We did not have any a priori expectations based on the participant demographics.

## Methods

### Study Design

The purpose of this analysis was to describe physician perspectives on addressing concerns regarding anti-Black racism, resolving conflicts, and facilitating accountability through use of a framework based on RJPs. The study followed the Consolidated Criteria for Reporting Qualitative Research (COREQ) reporting guideline. The University of Washington Institutional Review Board deemed the study exempt from review and the requirement of informed consent because the data were deidentified and the interview posed minimal risk to the participants.

### Participants

The methods have been described elsewhere.^19^ Briefly, we conducted one-on-one semistructured interviews 30 to 60 minutes in length with physicians at an academic county hospital in Seattle, Washington, between August 1 and October 31, 2022. A $20 gift card was provided to each of the physicians in appreciation of their participation.

### Interview Guide

Prior to the start of the interview, participants reported their age, gender, race and ethnicity, and years in practice. The interview included open-ended questions regarding patient experiences of racism in clinical settings and 2 scenarios in which anti-Black racism was an important contextual feature of a Black patient’s health care experience.^17^ These scenarios were taken directly from experiences shared by Black patients from a related qualitative study (eAppendix in Supplement 1). The first scenario focused on a patient’s concern of racism and undertreatment of pain. The second scenario focused on patient perceptions around discussions of emergency treatment options during a life-threatening event. In the second scenario, the patient’s diagnosis was modified to match a diagnosis with a median expected survival of 2 years or less in the participant’s subspecialty.^28^ After hearing each scenario, participants were asked to give their initial impressions, comment on how these scenarios resonated with them, and whether they had experienced similar interactions. They were asked what steps and resources would be needed to address the patient’s concerns and resolve conflict. If participants reported experiencing similar scenarios, they were asked to provide more details about that interaction, how the conflict was resolved, what went well, and what they wish could have gone better.

### Statistical Analysis

We describe researcher positionality, as beliefs, understandings, and lived experiences related to race and racism affect analyses and interpretation.^29,30^ One author (C.E.B.), a Black and Korean pulmonary and critical care physician trained in mixed methods research, conducted all interviews; she had prior professional, working relationships with 9 of the participants. Interviews were conducted past the point of thematic saturation for the purpose of the original study.^29^ With permission of the participants, interviews were audio-recorded, deidentified, and transcribed.

Two authors (C.E.B. and A.R.M. [a multiracial, Asian and Pacific Islander research coordinator with qualitative research experience]), conducted a second iteration of coding of all transcripts, producing a new list of codes. In alignment with RJPs, our coding framework used the following predefined themes: clinician-perpetrated harm, patient experiences of harms, clinician accountability, and reconciliation.^20,25^ Codes, their definitions, and their organization into each theme were reviewed by the research team including a Filipina American internal medicine resident with qualitative research experience (K.L.C.), and multiracial Black qualitative researcher (C.R.S.). Three authors (C.E.B., A.R.M., and K.L.C.) each triangulated coding by independently coding an initial set of 3 transcripts, then meeting to compare codes, identify similarities, reconcile discrepancies, and clarify definitions. Discrepancies were resolved through consensus. During initial stages of analyses, an additional theme, barriers to reconciliation, was added to the codebook. An additional 3 transcripts were independently coded by the team, with another meeting to review and compare the application of codes. The team coded the remaining transcripts with over half undergoing co-review by another member of the research team. Dedoose, version 9.0.86, was used to support the analyses.^31^

## Results

A total of 21 of 35 physicians (mean [SD] age, 44.2 [7.8] years) enrolled in the study, for a participation rate of 60.0% (Table 1). Participants were mostly women (14 [66.7%]), 4 were Asian (19.0%), 3 were Black (14.3%), and 14 were White (66.7%). Participants related to the scenarios, commenting that they were believable and easily facilitated discussions about anti-Black racism. The scenarios reminded many participants of prior patient and family interactions, prompting them to share personal experiences with patient concerns around anti-Black racism and conflict resolution processes that aligned with our restorative justice–based framework (Table 2).

**Table 1.** Participant Characteristics

Characteristic	Value (N = 21)
Age, mean (SD), y	44.2 (7.8)
Gender, No. (%)	
Women	14 (66.7)
Men	7 (33.3)
Years in practice as an attending physician, mean (SD)	10.8 (7.4)
Race, No. (%)	
Asian	4 (19.0)
Black	3 (14.3)
White	14 (66.7)
Specialty, No. (%)	
Pulmonary and critical care	8 (38.1)
Nephrology	5 (23.8)
Infectious disease	3 (14.3)
Gastroenterology	3 (14.3)
Cardiology	2 (9.5)
Academic rank, No. (%)	
Assistant professor	9 (42.9)
Associate professor	6 (28.6)
Professor	4 (19.0)

**Table 2.** Restorative Justice Themes and Representative Quotes

**Code**	**Definition**	**Quote**
**Theme 1: clinician-perpetuated harms**		
Harmful norms	Norms in clinical settings that are not always patient centered and may exacerbate perceptions of biases	“The fellow or the attending has an agenda or a framework they put on a conversation and the patient doesn’t feel listened to because they feel like they’re being put through a framework and not a conversation.”
Assuaging concerns	Reactive urge to explain or reassure patients that actions seen as biased or racist are harmless or unintentional	“A well-meaning team might feel like, ‘We’re going to go in there and reassure him.’ And the problem is he doesn’t feel like he’s listened to. He feels like he’s being steamrolled.”
Defensiveness	Actions and language by clinicians toward patients driven by defensiveness	“We’re all very quick to defend our decisions and reassure patients when they tell us we might be doing something that doesn’t feel good to them, why we’ve done it, and to defend why we’ve done it.”
**Theme 2: patient harms**		
Racialized health care experiences	Patients’ personal and vicarious experiences with racism that informs views about their care	“This has come up with my patients, stories of their childhood in which they’ve been denied care and they remember segregation and things like that. Even their adult children bring this up as well.”
Mistrust	Lack of trust in health care workers, technology, or health care institutions	“As part of the transplant workup, they do a mental health evaluation and ask, ‘Do you feel racism is a barrier?’ I’ve read from patients how they felt about providers, about being treated differently or a lack of trust.”
Intersectionality	Patients’ multiple marginalized identities exacerbate experiences of oppression	“I’ve had patients bring up they’re Black and some version of their identity whether its substance use or poverty or insurance status or various other things.”
**Theme 3: clinician accountability**		
Humility	Clinician willingness to be vulnerable, wrong, and to learn from their mistakes	“I mean, more education and humility building. Humility that we don’t know our minds as well as we think we do.”
Processing feedback	Finding time and space to process patient concerns before reacting	“I would like to have some time to reflect. One, am I doing this because of these reasons, and two, to have a thoughtful way to address it.”
Perspective taking	Pausing and considering patients’ views and lived experiences	“Patients have their own experiences and experiences of people in their community and you can’t remove the way they feel from that.”
**Theme 4: barriers to reconciliation**		
Misaligned values	A difference in clinician and patient values	“A plurality of values can create a moral distress. But it’s this evaluation that providers put on a patient, what value their life has. Can the provider set aside their values and recognize somebody else’s and honor them?”
Transparency	Patient and clinician hopes and concerns are not shared with one another	“I have cared for patients who have said, ‘I don’t understand what my treatment plan is’ or ‘No one is explaining it to me’ and ‘I’m worried that because of my race there are things that are being withheld.’”
**Theme 5: reconciliation**		
Mediators and advocates	Family member or clinician ensures that patients’ voices and concerns are heard and their choices are respected	“Ideally in a perfect world there would be all sorts of patient advocates.”
Validation	Clinicians actively listen to patients, and patients’ concerns are heard, validated, and normalized	“When I’ve seen [conflict] navigated well is when folks take the time to hear the patient, respond with honesty, and validate their concerns.”
Mutual understanding	After understanding the other’s perspective, a patient and clinician come to agreement about a plan of care aligned with the patient’s values	“I build rapport, learn about their experience, their ups and downs, values, and hopes. Let them tell their story. I add to the discussion based on what I’m hearing.”
Patient centeredness	Conversations and plan of care focus on patient’s concerns and values; patient is given additional knowledge, strategy, and choice going forward	“‘Tell me your concerns, tell me about the things that we’re not doing for you, about what you’re worried about.’ It’s a lot of asking to get a sense of how I might give answers to address the core concerns.”

### Clinician-Perpetrated Harm

Participants reflected on norms that may exacerbate perceptions about bias (Table 2). For example, the practice of repeatedly confirming and readdressing a patient’s preferences for emergency treatment options during a life-threatening event as a patient became progressively more ill or changed services was seen as well meaning but potentially harmful. Readdressing the patient’s preferences for emergency treatment options was seen as detrimental, especially when discussions were prompted by expectations from others caring for the patient and not by a patient-centered approach. One participant stated, “Trainees feel a lot of pressure to work out the [patient’s preferences for emergency treatment options] from the care team.” In addition, participants widely reported the presence of bias in the initiation and revisitation of patient preferences for emergency treatment options, noting that these discussions, “may be informed by biases, just like when we question someone’s decisional capacity.” Participants noted they and others wanted to assuage patient concerns about bias by reassuring patients and families, but they often did not address core concerns about bias and racism. Attempts to alleviate concerns about bias were often accompanied by defensiveness, especially when it was implied that they or their team were racist. One participant noted, “We’re quick to say race has nothing to do with it and people just don’t believe us.” Another shared, “So much of how we communicate with patients is defensive.”

### Patient Experiences of Harm

Participants described being aware that Black patients experience racialized health care, with both personal and vicarious experiences informing patients’ views (Table 2). One participant noted, “Studies show pain medications are not prescribed the same way. [The patient] could have heard this before. If he’s still suffering from pain, I could easily see him wondering.” Some participants were aware of stereotype threat, Black patients’ concerns about conforming to negative racial stereotypes, especially if they had personally experienced being stereotyped or profiled^32^: “[The patient’s] concern about appearing as an angry Black man is understandable. They’re gonna [sic] think he’s drug seeking.” Participants felt mistrust and suspicion were common among Black patients, especially during conflict around medical decision-making. One participant shared their experience when attempting to discuss preferences for emergency treatment options with a Black patient: “He didn’t say, ‘Is it because I’m Black?’ But you could tell he was thinking it.” Participants recognized that having multiple marginalized identities, such as experiencing addiction or being unhoused, exacerbated bias and prompted value judgements on their quality of life: “We go, ‘This patient’s quality of life is not great, they would not do well with CPR [cardiopulmonary resuscitation], so let me ask [about preferences for emergency treatment options] to see if I get an appropriate response.’ That happens a lot.”

### Clinician Accountability

Participants observed that usual attempts to address concerns about anti-Black racism were ineffective, and instead emphasized active listening as an often missed but important first step (Table 2). Participants noted that actively listening while resisting the urge to react and defend oneself was challenging, but necessary. One participant shared, “To step outside yourself and really see the situation is really hard without digging in.” Participants reported that time and space to reflect and process their role in perpetuating racism was limited, but important to help facilitate perspective-taking. By taking time to reflect, participants noted that they were better able to recognize a patient’s perspectives about racist care, regardless of whether they were receiving standard, evidence-based care or not: “Let’s recognize that there’s many reasons why patients could be feeling this way. Maybe they are getting the right treatment and it doesn’t invalidate the fact that they’re concerned.”

### Addressing Barriers to Reconciliation

Participants noted clinician values are frequently misaligned with patients’ values, often resulting in negative perceptions of patient and family choices (Table 2). These conflicting values contributed to the moral distress participants experienced and worsened perceptions and communication with patients when there was conflict. One participant shared, “Our values are not aligned with what patients need, especially patients with social barriers and challenges to receiving health care.” Participants believed improved transparency from both clinicians and patients might improve understanding around goals of care and help find alignment. One participant shared, “I wonder if a patient or their family are not feeling like they can bring up what it is that is keeping them from making certain choices.” Another participant shared, “I tell patients and families that I come to this with my own set of values and that’s part of where our guidance comes from. I think that that has been positively received.”

### Reconciliation

Participants noted that creating an environment in which patients felt comfortable sharing their thoughts in the presence of a trusted HCW or advocate was important (Table 2). Many thought an advocate would also help prompt clinician self-reflection and provide “additional support for the patient, someone that could be an ally.” The presence of mediators and advocates was endorsed by participants as helpful resources to guide clinicians toward validating and normalizing patients’ concerns. After reflecting on the scenarios, a participant stated, “Getting someone else to help assess the situation would help with that additional perspective of, have I really offered the same to this patient as other patients?” Participants thought advocates would help move clinicians away from defensive behaviors and language and facilitate a patient-focused approach that validated concerns and facilitated mutual understanding: “There’s a gap in communication that needs to be addressed for both parties to understand where the other is coming from and find alignment.” Mutual understanding facilitates a patient-centered approach to finding a plan that is acceptable to the patient, even if this plan is not in line with a clinician’s values. For many participants, this meant focusing on the patient’s goals and the processes needed to achieve them. One participant shared, “What I bring to the discussion is based on what I’m hearing from [the patient] so at least the patient is initiating. They’re given the first volley of saying, ‘This is what’s important to me,’ and then I can say, ‘Well hearing that, it sounds like this is how we should take care of you.’” Last, only 2 participants offered apologies as a part of reconciliation. One participant stated, “Hopefully there’s going to be an apology and a step about what you’re going to do differently next time.”

## Discussion

In this study, we describe skills and resources identified by physicians to address the effect and harm of anti-Black racism in clinical settings through the novel use of a restorative justice framework to guide qualitative analyses around communication about serious illness. Through the combined use of provided scenarios and personal experiences, participants described harmful behaviors and cultural norms that perpetuate and exacerbate anti-Black racism in the hospital setting, even if these harms are unintentional. Participants identified potential steps to resolve conflict and address patient concerns, but additional processes are needed to facilitate repair and accountability. Prior reports of the application of RJPs in schools suggest that these practices are associated with a decrease in harmful, biased disciplinary practices against students of racial and ethnic minority populations, lower levels of absenteeism, and an increased sense of community.^33^ Studies suggest that educators benefit from RJPs, but learning and accepting restorative approaches require a transformative shift away from status quo concepts of accountability that protects privilege and social standing.^34,35^ Similarly, actualizing accountability for anti-Black racism in health care settings requires shifting away from the power and privilege afforded to HCWs and health care institutions, and toward patients and communities that are traditionally marginalized.^36,37,38^ This suggests a need to introduce and educate stakeholders about RJPs before broad implementation.

The use of scenarios in this study promoted discussions with participants about conflict perpetuated by anti-Black racism and the difficulties resolving them, enabling us to expand on findings in the original study. For example, participants described how cultural norms and usual communication practices may facilitate biased behavior or result in misunderstandings that further marginalize patients. Although the original study described initial feelings of defensiveness on hearing patients’ concerns about racism in their care, a restorative justice lens further illuminated how defensive behaviors and language continue to propagate more harm on marginalized patients. The concepts of time and space in the original study were described as a need that mostly served physicians to help them process hard-to-hear feedback; with a restorative justice framework, time and space further emerged as important components to facilitate perspective taking, validate patient concerns, decrease harmful behaviors, and promote mutual understanding to find a path forward in a plan of care. Transparency in the original study was largely described as a factor to bring awareness to clinicians about patients’ concerns, but a restorative justice lens suggests that transparency is needed from both patients and clinicians, as the incongruency of clinicians’ values with those of their patients contributed to moral distress and worsened patient-clinician conflict.

Participants in the original analyses shared that patients should feel empowered to bring their concerns about racism and other inequities to clinicians or other HCWs. However, our findings suggest that common communication practices, especially those rooted in defensiveness, may result in more harm to patients if concerns about racism are shared. Prior studies suggest that poor communication skills and the minimization of family voices contributes to and worsens conflict.^39,40,41^ The presence of unconscious bias was widely reported by participants, but only a handful of participants noted biases of their own. In addition, while participants in our study agreed that managing defensiveness and partaking in active listening was important, most stopped short of actualizing responsibility for their actions. Restorative justice processes may mitigate harmful pedagogical practices, improve communication processes, and promote accountability when Black patients and others who are marginalized have been harmed.^20,42,43^

### Limitations

There are some limitations in our study. First, this is a single-center study with a small number of participants, which may limit transferability of our findings. Physicians in our study may have differing views from those who did not respond to requests for participation and participants with a working relationship with the interviewer may have been more willing to be open and forthcoming about their experiences and perspectives. In addition, this was a secondary qualitative analysis and thematic saturation was defined for the original qualitative study. Most participants were White, and no advanced practice professionals were included. In addition, the perspectives of medical learners were not captured in this study.

## Conclusions

Physicians identified resources, skills, and processes that partially aligned with a restorative justice framework to address the harms of anti-Black racism, but additional steps are needed to actualize accountability and facilitate racial healing. Current communication practices may perpetuate more harm, but RJPs may help resolve conflict during shared decision-making processes and facilitate racial healing when patients and families have been harmed by anti-Black racism.

## Notes

Supplement 1. **eAppendix.** Scenarios and Interview Guide

Supplement 2.
Data Sharing Statement
