# Supplementary material for: Physician Perspectives on Addressing Anti-Black Racism
Source: JAMA Netw Open. 2024 Jan 24;7(1):e2352818. doi: 10.1001/jamanetworkopen.2023.52818 (PMC10809013; doi:10.1001/jamanetworkopen.2023.52818)
Supplement: Supplement 1. — eAppendix. Scenarios and Interview Guide [file jamanetwopen-e2352818-s001.pdf]

Supplementary Online Content

Brown CE, Marshall AR, Cueva KL, Snyder CR, Kross EK, Young BA. Physician perspectives on addressing anti-Black racism. *JAMA Netw Open.* 2024;7(1):e2352818. doi:10.1001/jamanetworkopen.2023.52818

eAppendix. Scenarios and Interview Guide

This supplementary material has been provided by the authors to give readers additional information about their work.

© 2024 Brown CE et al. *JAMA Network Open.*

eAppendix. Scenarios and Interview Guide

<u>Scenario 1:</u> Mr. Evans is a 64-year-old Black man with metastatic cancer. He is upset and feels that his pain is not being properly treated. He also thinks other treatment modalities are being withheld from him though he cannot say what exactly. He believes he is being mistreated because he is Black and doesn't have a lot of money. He wants to talk to his doctors more about this, but every time he brings this up, he feels his treatment team is too quick to try to reassure him. He is worried about appearing as an angry Black man, especially since his treating team is made up of all White physicians.

<u>Scenario 2:</u> Mrs. Johnson is a 59-year-old Black woman with *[provider specialty specific diagnosis].* She has been admitted multiple times for COPD exacerbations. Each time she is admitted, her care team attempts to discuss her goals of care and whether or not she would like to be intubated. Mrs. Johnson wants to be intubated and has always wanted CPR and wondering doctors continue to ask this question because they do not want to her to live because she is Black and has a history of using illicit substances in the past. While she is curious about this, she doesn't ask about it because she is afraid that her doctors and nurses will retaliate against her by withholding more treatment.

**Suggested prompt depending on provider specialty for scenario #2**	
<u>Specialty</u>	<u>Diagnosis</u>
Pulmonary and Critical Care	*COPD on home oxygen*
Cardiology	*NYHA class III heart failure*
Infectious Disease	*Stage 3 HIV*
Nephrology	*Diabetes and ESRD*
Gastroenterology	*Child C cirrhosis*

© 2024 Brown CE et al. *JAMA Network Open.*

After each scenario:

- What's your first reaction when you hear this? What comes to mind?
- Have you been in a situation like this before?
    - How did you navigate it?
    - Was there anything that you wish you did differently?
    - What, if any, resources do you wish you had to help navigate this situation?
- What tool or skill would you like to have to help talk with a patient about racist healthcare experiences that involved you or your team.
    - What would make you feel prepared to support or engage with that patient?
    - What would make you or your team feel supported?

© 2024 Brown CE et al. *JAMA Network Open.*
