# Supplementary material for: Physician Perspectives on Addressing Anti-Black Racism
Source: JAMA Netw Open. 2024 Jan 24;7(1):e2352818. doi: 10.1001/jamanetworkopen.2023.52818 (PMC10809013; doi:10.1001/jamanetworkopen.2023.52818)
Supplement: Supplement 2. — Data Sharing Statement [file jamanetwopen-e2352818-s002.pdf]

Data Sharing Statement

Brown. Physician Perspectives on Addressing Anti-Black Racism. *JAMA Netw Open*. Published January 24, 2024. doi:10.1001/jamanetworkopen.2023.52818

Data
Data available: No

Additional Information
Explanation for why data not available: This data will not be shared due to the sensitive nature of these data.
